# 1,1-Dimethyl­hydrazin-1-ium picrate

**DOI:** 10.1107/S1600536811038554

**Published:** 2011-09-30

**Authors:** Xiao-Gang Mu, Xuan-Jun Wang, Xiang-Xuan Liu, Hu Cui, Huanchun Wang

**Affiliations:** a No. 503 Faculty, Xi’an Research Institute of High Technology, Hongqing Town, Xi’an 710025, People’s Republic of China

## Abstract

In the title compound, C_2_H_9_N_2_^+^·C_6_H_2_N_3_O_7_^−^, the dihedral angles between the mean planes of the three nitro groups and the benzene ring are 63.5 (3), 10.5 (2) and 10.4 (2)°. In the crystal, mol­ecules are linked by N—H⋯O hydrogen bonds into a two-dimensional network parallel to (001).

## Related literature

For related structures, see: Merkoulov *et al.* (2005 ▶); Yang *et al.* (2002 ▶).
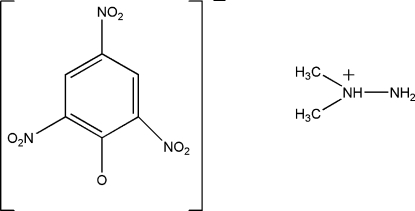


## Experimental

### 

#### Crystal data

C_2_H_9_N_2_^+^·C_6_H_2_N_3_O_7_^−^
*M* *_r_* = 289.22
Monoclinic, 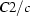

*a* = 14.2038 (17) Å
*b* = 8.1932 (10) Å
*c* = 21.233 (3) Å
β = 98.298 (2)°
*V* = 2445.1 (5) Å^3^
*Z* = 8
Mo *K*α radiation
μ = 0.14 mm^−1^
*T* = 296 K
0.37 × 0.25 × 0.15 mm

#### Data collection

Bruker APEXII CCD diffractometer
Absorption correction: multi-scan (*SADABS*; Sheldrick, 1996 ▶) *T*_min_ = 0.951, *T*_max_ = 0.980
6952 measured reflections
2781 independent reflections
1878 reflections with *I* > 2σ(*I*)
*R*_int_ = 0.035

#### Refinement

*R*[*F* ^2^ > 2σ(*F* ^2^)] = 0.055
*wR*(*F* ^2^) = 0.135
*S* = 1.08
2781 reflections
195 parameters
H atoms treated by a mixture of independent and constrained refinement
Δρ_max_ = 0.36 e Å^−3^
Δρ_min_ = −0.39 e Å^−3^

### 

Data collection: *APEX2* (Bruker, 2007 ▶); cell refinement: *SAINT* (Bruker, 2007 ▶); data reduction: *SAINT*; program(s) used to solve structure: *SHELXS97* (Sheldrick, 2008 ▶); program(s) used to refine structure: *SHELXL97* (Sheldrick, 2008 ▶); molecular graphics: *PLATON* (Spek, 2009 ▶); software used to prepare material for publication: *SHELXTL* (Sheldrick, 2008 ▶).

## Supplementary Material

Crystal structure: contains datablock(s) global, I. DOI: 10.1107/S1600536811038554/lh5325sup1.cif

Structure factors: contains datablock(s) I. DOI: 10.1107/S1600536811038554/lh5325Isup2.hkl

Supplementary material file. DOI: 10.1107/S1600536811038554/lh5325Isup3.cml

Additional supplementary materials:  crystallographic information; 3D view; checkCIF report

## Figures and Tables

**Table 1:** Hydrogen-bond geometry (Å, °)

*D*—H⋯*A*	*D*—H	H⋯*A*	*D*⋯*A*	*D*—H⋯*A*
N5—H5*A*⋯O7	0.86 (3)	2.15 (3)	2.964 (3)	158 (3)
N5—H5*B*⋯O1^i^	0.85 (3)	2.55 (3)	3.308 (3)	150 (2)
N4—H7*D*⋯O7^ii^	0.90 (2)	1.84 (2)	2.694 (2)	157 (2)
N4—H7*D*⋯O6^ii^	0.90 (2)	2.36 (2)	2.924 (2)	120.3 (18)

Symmetry codes: (i) 
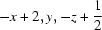
; (ii) 
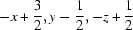
.

**Fig. 1.**
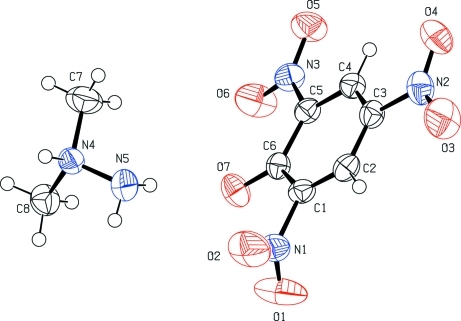
The molecular structure of the title compound showing 50% probability displacement ellipsoids.

## supplementary crystallographic information

### Comment

The molecular structure of the title compound is shown in Fig. 1. Examples of related structures have already been published (Merkoulov *et al.*, 2005; Yang *et al.*, 2002).

### Experimental

1,1-dimethylhydrazine (0.02 mol) was added to a solution of picric acid (0.02 mol) in 30 ml ethanol at room temperature, the mixture was stirred for 0.5 h to afford the title compound. Single crystals suitable for X-ray structure analysis were obtained by slowly evaporating a distilled water solution of the title compound at room temperature.

### Refinement

H atoms bonded to C atoms were fixed geometrically and allowed to ride on their attached atoms, with C—H distances = 0.93–0.96 Å and with *U*_iso_(H) = 1.2*U*_eq_(C) or 1.5*U*_eq_(C_methyl_). H atoms bonded to N atoms were refined independently with isotropic displacement parameters.

### Crystal data

**Table:**

C_2_H_9_N_2_^+^·C_6_H_2_N_3_O_7_^−^	*F*(000) = 1200
*M**_r_* = 289.22	*D*_x_ = 1.571 Mg m^−^^3^
Monoclinic, *C*2/*c*	Mo *K*α radiation, λ = 0.71073 Å
Hall symbol: -C 2yc	Cell parameters from 1401 reflections
*a* = 14.2038 (17) Å	θ = 2.9–24.3°
*b* = 8.1932 (10) Å	µ = 0.14 mm^−^^1^
*c* = 21.233 (3) Å	*T* = 296 K
β = 98.298 (2)°	Block, yellow
*V* = 2445.1 (5) Å^3^	0.37 × 0.25 × 0.15 mm
*Z* = 8	

### Data collection

**Table:**

Bruker APEXII CCD diffractometer	2781 independent reflections
Radiation source: fine-focus sealed tube	1878 reflections with *I* > 2σ(*I*)
graphite	*R*_int_ = 0.035
φ and ω scans	θ_max_ = 27.6°, θ_min_ = 2.9°
Absorption correction: multi-scan (*SADABS*; Sheldrick, 1996)	*h* = −9→18
*T*_min_ = 0.951, *T*_max_ = 0.980	*k* = −10→10
6952 measured reflections	*l* = −27→26

### Refinement

**Table:**

Refinement on *F*^2^	Secondary atom site location: difference Fourier map
Least-squares matrix: full	Hydrogen site location: inferred from neighbouring sites
*R*[*F*^2^ > 2σ(*F*^2^)] = 0.055	H atoms treated by a mixture of independent and constrained refinement
*wR*(*F*^2^) = 0.135	*w* = 1/[σ^2^(*F*_o_^2^) + (0.0553*P*)^2^ + 0.8977*P*] where *P* = (*F*_o_^2^ + 2*F*_c_^2^)/3
*S* = 1.08	(Δ/σ)_max_ = 0.002
2781 reflections	Δρ_max_ = 0.36 e Å^−^^3^
195 parameters	Δρ_min_ = −0.39 e Å^−^^3^
0 restraints	Extinction correction: *SHELXL*, Fc^*^=kFc[1+0.001xFc^2^λ^3^/sin(2θ)]^-1/4^
Primary atom site location: structure-invariant direct methods	Extinction coefficient: 0.0133 (15)

### Special details

**Table:**

Geometry. All e.s.d.'s (except the e.s.d. in the dihedral angle between two l.s. planes) are estimated using the full covariance matrix. The cell e.s.d.'s are taken into account individually in the estimation of e.s.d.'s in distances, angles and torsion angles; correlations between e.s.d.'s in cell parameters are only used when they are defined by crystal symmetry. An approximate (isotropic) treatment of cell e.s.d.'s is used for estimating e.s.d.'s involving l.s. planes.
Refinement. Refinement of *F*^2^ against ALL reflections. The weighted *R*-factor *wR* and goodness of fit *S* are based on *F*^2^, conventional *R*-factors *R* are based on *F*, with *F* set to zero for negative *F*^2^. The threshold expression of *F*^2^ > σ(*F*^2^) is used only for calculating *R*-factors(gt) *etc*. and is not relevant to the choice of reflections for refinement. *R*-factors based on *F*^2^ are statistically about twice as large as those based on *F*, and *R*- factors based on ALL data will be even larger.

### Fractional atomic coordinates and isotropic or equivalent isotropic displacement parameters (Å^2^)

**Table:**

	*x*	*y*	*z*	*U*_iso_*/*U*_eq_	
O1	1.04883 (15)	0.9752 (3)	0.34381 (11)	0.0893 (7)	
O2	1.00739 (14)	0.7278 (2)	0.33165 (10)	0.0805 (6)	
O3	1.00992 (13)	0.6369 (2)	0.57533 (9)	0.0771 (6)	
O4	0.87633 (13)	0.7067 (2)	0.60489 (8)	0.0606 (5)	
O5	0.67812 (11)	1.0875 (2)	0.48554 (8)	0.0562 (4)	
O6	0.71397 (12)	1.2026 (2)	0.40207 (9)	0.0680 (5)	
O7	0.84917 (10)	1.06520 (17)	0.34354 (6)	0.0462 (4)	
N1	1.00342 (12)	0.8594 (2)	0.35666 (9)	0.0451 (5)	
N2	0.93391 (14)	0.7096 (2)	0.56715 (9)	0.0483 (5)	
N3	0.72977 (12)	1.1019 (2)	0.44486 (9)	0.0408 (4)	
C1	0.94420 (13)	0.8768 (2)	0.40738 (9)	0.0344 (4)	
C2	0.96717 (13)	0.7855 (2)	0.46090 (9)	0.0371 (5)	
H2	1.0187	0.7143	0.4652	0.044*	
C3	0.91097 (13)	0.8022 (2)	0.50884 (9)	0.0361 (5)	
C4	0.83413 (13)	0.9051 (2)	0.50215 (10)	0.0365 (4)	
H4	0.7965	0.9127	0.5344	0.044*	
C5	0.81262 (13)	0.9972 (2)	0.44776 (9)	0.0332 (4)	
C6	0.86629 (13)	0.9897 (2)	0.39543 (9)	0.0326 (4)	
N4	0.69915 (12)	0.8697 (2)	0.19680 (8)	0.0378 (4)	
N5	0.78695 (15)	0.8351 (3)	0.23798 (11)	0.0506 (5)	
H5A	0.7935 (19)	0.919 (3)	0.2626 (14)	0.074 (9)*	
H5B	0.8288 (18)	0.831 (3)	0.2133 (13)	0.064 (8)*	
C7	0.62138 (17)	0.8862 (3)	0.23556 (13)	0.0603 (7)	
H7A	0.6170	0.7882	0.2598	0.090*	
H7B	0.5624	0.9040	0.2081	0.090*	
H7C	0.6342	0.9771	0.2640	0.090*	
C8	0.70463 (16)	1.0116 (3)	0.15431 (10)	0.0478 (5)	
H8A	0.7128	1.1096	0.1793	0.072*	
H8B	0.6469	1.0189	0.1247	0.072*	
H8C	0.7577	0.9983	0.1314	0.072*	
H7D	0.6867 (16)	0.779 (3)	0.1733 (12)	0.054 (7)*	

### Atomic displacement parameters (Å^2^)

**Table:**

	*U*^11^	*U*^22^	*U*^33^	*U*^12^	*U*^13^	*U*^23^
O1	0.0967 (15)	0.0698 (13)	0.1180 (18)	−0.0159 (11)	0.0723 (13)	−0.0003 (12)
O2	0.0916 (14)	0.0776 (13)	0.0813 (14)	−0.0073 (11)	0.0424 (11)	−0.0351 (11)
O3	0.0667 (12)	0.0918 (14)	0.0690 (13)	0.0318 (11)	−0.0025 (9)	0.0300 (11)
O4	0.0776 (12)	0.0632 (11)	0.0426 (10)	−0.0001 (9)	0.0139 (9)	0.0113 (8)
O5	0.0470 (9)	0.0668 (11)	0.0589 (10)	0.0182 (8)	0.0216 (8)	0.0022 (8)
O6	0.0584 (10)	0.0697 (11)	0.0795 (13)	0.0305 (9)	0.0219 (9)	0.0322 (10)
O7	0.0608 (9)	0.0421 (8)	0.0361 (8)	0.0118 (7)	0.0081 (7)	0.0045 (6)
N1	0.0369 (10)	0.0530 (11)	0.0471 (11)	0.0065 (8)	0.0113 (8)	−0.0010 (9)
N2	0.0564 (12)	0.0457 (10)	0.0399 (11)	0.0027 (9)	−0.0020 (9)	0.0055 (8)
N3	0.0350 (9)	0.0401 (9)	0.0479 (11)	0.0073 (7)	0.0073 (8)	0.0004 (8)
C1	0.0291 (9)	0.0358 (10)	0.0393 (11)	−0.0001 (8)	0.0084 (8)	−0.0041 (8)
C2	0.0303 (10)	0.0358 (10)	0.0437 (12)	0.0031 (8)	0.0004 (8)	−0.0007 (9)
C3	0.0355 (10)	0.0353 (10)	0.0359 (11)	0.0012 (8)	−0.0008 (8)	0.0032 (8)
C4	0.0341 (10)	0.0401 (11)	0.0355 (11)	−0.0017 (8)	0.0063 (8)	−0.0033 (9)
C5	0.0287 (9)	0.0324 (10)	0.0382 (11)	0.0033 (7)	0.0041 (8)	−0.0019 (8)
C6	0.0348 (10)	0.0297 (9)	0.0326 (10)	−0.0012 (8)	0.0018 (8)	−0.0027 (8)
N4	0.0408 (10)	0.0391 (9)	0.0338 (10)	−0.0045 (7)	0.0068 (7)	−0.0019 (8)
N5	0.0497 (12)	0.0603 (13)	0.0397 (12)	−0.0013 (10)	−0.0004 (9)	0.0047 (10)
C7	0.0552 (14)	0.0719 (16)	0.0595 (16)	−0.0023 (12)	0.0277 (12)	−0.0014 (13)
C8	0.0555 (13)	0.0428 (12)	0.0443 (13)	−0.0024 (10)	0.0052 (10)	0.0033 (10)

### Geometric parameters (Å, °)

**Table:**

O1—N1	1.201 (2)	C4—C5	1.376 (3)
O2—N1	1.207 (2)	C4—H4	0.9300
O3—N2	1.223 (2)	C5—C6	1.437 (3)
O4—N2	1.225 (2)	N4—N5	1.444 (3)
O5—N3	1.217 (2)	N4—C7	1.476 (3)
O6—N3	1.224 (2)	N4—C8	1.480 (3)
O7—C6	1.256 (2)	N4—H7D	0.90 (2)
N1—C1	1.466 (2)	N5—H5A	0.86 (3)
N2—C3	1.449 (3)	N5—H5B	0.85 (3)
N3—C5	1.450 (2)	C7—H7A	0.9600
C1—C2	1.360 (3)	C7—H7B	0.9600
C1—C6	1.437 (3)	C7—H7C	0.9600
C2—C3	1.388 (3)	C8—H8A	0.9600
C2—H2	0.9300	C8—H8B	0.9600
C3—C4	1.370 (3)	C8—H8C	0.9600
			
O1—N1—O2	123.0 (2)	O7—C6—C5	126.88 (17)
O1—N1—C1	118.33 (18)	O7—C6—C1	121.24 (17)
O2—N1—C1	118.55 (18)	C5—C6—C1	111.84 (16)
O3—N2—O4	123.84 (19)	N5—N4—C7	109.31 (19)
O3—N2—C3	117.47 (19)	N5—N4—C8	113.98 (17)
O4—N2—C3	118.69 (18)	C7—N4—C8	112.17 (18)
O5—N3—O6	121.83 (17)	N5—N4—H7D	104.9 (15)
O5—N3—C5	118.77 (17)	C7—N4—H7D	106.4 (15)
O6—N3—C5	119.39 (17)	C8—N4—H7D	109.6 (15)
C2—C1—C6	125.98 (17)	N4—N5—H5A	102.7 (19)
C2—C1—N1	117.82 (17)	N4—N5—H5B	104.7 (18)
C6—C1—N1	116.20 (17)	H5A—N5—H5B	112 (3)
C1—C2—C3	117.66 (17)	N4—C7—H7A	109.5
C1—C2—H2	121.2	N4—C7—H7B	109.5
C3—C2—H2	121.2	H7A—C7—H7B	109.5
C4—C3—C2	121.25 (18)	N4—C7—H7C	109.5
C4—C3—N2	119.24 (18)	H7A—C7—H7C	109.5
C2—C3—N2	119.52 (17)	H7B—C7—H7C	109.5
C3—C4—C5	120.08 (18)	N4—C8—H8A	109.5
C3—C4—H4	120.0	N4—C8—H8B	109.5
C5—C4—H4	120.0	H8A—C8—H8B	109.5
C4—C5—C6	123.18 (17)	N4—C8—H8C	109.5
C4—C5—N3	116.11 (17)	H8A—C8—H8C	109.5
C6—C5—N3	120.69 (17)	H8B—C8—H8C	109.5
			
O1—N1—C1—C2	−115.2 (2)	C3—C4—C5—C6	1.2 (3)
O2—N1—C1—C2	61.8 (3)	C3—C4—C5—N3	179.71 (17)
O1—N1—C1—C6	65.0 (3)	O5—N3—C5—C4	−9.2 (3)
O2—N1—C1—C6	−118.1 (2)	O6—N3—C5—C4	170.30 (18)
C6—C1—C2—C3	−0.2 (3)	O5—N3—C5—C6	169.29 (18)
N1—C1—C2—C3	179.97 (17)	O6—N3—C5—C6	−11.2 (3)
C1—C2—C3—C4	1.0 (3)	C4—C5—C6—O7	177.27 (18)
C1—C2—C3—N2	−178.88 (17)	N3—C5—C6—O7	−1.1 (3)
O3—N2—C3—C4	−170.0 (2)	C4—C5—C6—C1	−0.5 (3)
O4—N2—C3—C4	10.8 (3)	N3—C5—C6—C1	−178.87 (16)
O3—N2—C3—C2	9.9 (3)	C2—C1—C6—O7	−177.91 (18)
O4—N2—C3—C2	−169.33 (19)	N1—C1—C6—O7	1.9 (3)
C2—C3—C4—C5	−1.5 (3)	C2—C1—C6—C5	0.0 (3)
N2—C3—C4—C5	178.38 (17)	N1—C1—C6—C5	179.75 (16)

### Hydrogen-bond geometry (Å, °)

**Table:**

*D*—H···*A*	*D*—H	H···*A*	*D*···*A*	*D*—H···*A*
N5—H5A···O7	0.86 (3)	2.15 (3)	2.964 (3)	158 (3)
N5—H5B···O1^i^	0.85 (3)	2.55 (3)	3.308 (3)	150 (2)
N4—H7D···O7^ii^	0.90 (2)	1.84 (2)	2.694 (2)	157 (2)
N4—H7D···O6^ii^	0.90 (2)	2.36 (2)	2.924 (2)	120.3 (18)

Symmetry codes: (i) −*x*+2, *y*, −*z*+1/2; (ii) −*x*+3/2, *y*−1/2, −*z*+1/2.
